# Case-case study on comparative vaccine effectiveness against Delta and Omicron SARS-CoV-2 infections: Irina Kislaya

**DOI:** 10.1093/eurpub/ckac129.668

**Published:** 2022-10-25

**Authors:** I Kislaya, A Peralta-Santos, L Vieira, C Sousa, B Ferreira, A Pelerito, JP Gomes, P Pinto Leite, B Nunes, PT COVID-19 group

**Affiliations:** 1 Departamento de Epidemiologia, National Institute of Health Doutor Ricardo Jorge, Lisbon, Portugal; 2 Public Health Research Centre, Universidade NOVA de Lisboa, Lisbon, Portugal; 3 Comprehensive Health Research Centre, Universidade NOVA de Lisboa, Lisbon, Portugal; 4 Direção de Serviços de Informação e Análise, Direção-Geral da Saúde, Lisbon, Portugal; 5 Department of Human Genetics, National Institute of Health Doutor Ricardo Jorge, Lisbon, Portugal; 6 Unilabs, Porto, Portugal; 7 Algarve Biomedical Center Research Institute, Faro, Portugal; 8 Faculty of Medicine and Biomedical Sciences, University of Algarve, Faro, Portugal; 9 Portuguese Red Cross Laboratory, Lisbon, Portugal; 10 Department of Infectious Diseases, National Institute of Health Doutor Ricardo Jorge, Lisbon, Portugal

## Abstract

**Introduction:**

Vaccination is the primary pharmacological measure to reduce SARS-CoV-2 transmission and its complications. Timely information on vaccines effectiveness in a context of novel variants of concern (VOC) emergence is essential for public health policies. This study aimed to provide a measure of comparative vaccine effectiveness between Omicron (BA.1) and Delta (B.1.627.2) VOC according to vaccination exposure (complete primary regimen or booster dose) for Portuguese population aged 12 or more years old using routinely collected data from electronic health records.

**Methods:**

We used a case-case study design linking national electronic vaccination registry and surveillance data on 13,134 SARS-CoV-2 RT-PCR laboratory-confirmed cases notified in Portugal during weeks 49-51 of 2021. Notified cases were classified as Omicron or Delta based on whole-genome sequencing or S-gene Target Failure (SGTF) status using the RT-PCR TaqPath™ Covid 19 CE IVD Kit (Thermo Scientific™) assay. The odds of vaccination was compared between Omicron cases and Delta cases using logistic regression adjusted for age group, sex, region and week of diagnosis and laboratory of origin.

**Results:**

The odds of vaccination were higher in laboratory-confirmed cases infected by Omicron (BA.1) VOC compared to Delta (B.1.627.2) VOC for both complete primary vaccination (Odds ratio (OR)=2.1; 95% Confidence Interval (95%CI): 1.8 - 2.4) and booster dose (OR = 5.2; 95%CI: 3.1 - 8.8), indicating vaccine effectiveness reduction against Omicron.

**Conclusions:**

We found significantly higher odds of vaccination in Omicron cases compared to Delta, suggesting lower effectiveness of the primary vaccination and the booster dose in preventing infections by Omicron. Case-case study design has proven to be feasible approach to rapidly compare vaccine effectiveness between VOC in context of novel VOC emergence to timely inform public health stakeholders.

**Key messages:**

• Reduction of vaccine-induced protection against SARS-COV-2 infection with the Omicron compared to Delta after primary and booster vaccination.

• Continuous monitoring of COVID-19 vaccine effectiveness is essential to support public health policies in context of novel VOC emergence.

